# Lenses and levels: the why, what and how of measuring health system drivers of women’s, children’s and adolescents’ health with a governance focus

**DOI:** 10.1136/bmjgh-2018-001316

**Published:** 2019-06-24

**Authors:** Asha George, Amnesty Elizabeth LeFevre, Tanya Jacobs, Mary Kinney, Kent Buse, Mickey Chopra, Bernadette Daelmans, Annie Haakenstad, Luis Huicho, Rajat Khosla, Kumanan Rasanathan, David Sanders, Neha S Singh, Nicki Tiffin, Rajani Ved, Shehla Abbas Zaidi, Helen Schneider

**Affiliations:** 1 School of Public Health, University of the Western Cape, Cape Town, South Africa; 2 School of Public Health and Family Medicine, University of CapeTown, Cape Town, Maryland; 3 Political Affairs and Strategy, UNAIDS, Geneva, Switzerland; 4 World Bank, Washington, District of Columbia, USA; 5 World Health Organisation, Geneva, Switzerland; 6 School of Public Health, Harvard University, Boston, Massachusetts, USA; 7 Universidad Peruana Cayetano Heredia, Lima, Peru; 8 United Nations High Commission for Human Rights, Geneva, Switzerland; 9 World Health Organisation, Phnom Penh, Cambodia; 10 Department of Global Health and Development, London School of Hygiene and Tropical Medicine, London, UK; 11 Centre for Infectious Disease Research in Africa, University of Cape Town, Cape Town, South Africa; 12 Computational Biology, University of Cape Town, Cape Town, South Africa; 13 National Health Systems Resource Centre, New Delhi, India; 14 Community Health Sciences, Aga Khan University Faculty of Health Sciences, Karachi, Pakistan

**Keywords:** health systems, governance, measurement, rights, power, epistemology

## Abstract

Health systems are critical for health outcomes as they underpin intervention coverage and quality, promote users’ rights and intervene on the social determinants of health. Governance is essential for health system endeavours as it mobilises and coordinates a multiplicity of actors and interests to realise common goals. The inherently social, political and contextualised nature of governance, and health systems more broadly, has implications for measurement, including how the health of women, children and adolescents health is viewed and assessed, and for whom. Three common lenses, each with their own views of power dynamics in policy and programme implementation, include a service delivery lens aimed at scaling effective interventions, a societal lens oriented to empowering people with rights to effect change and a systems lens concerned with creating enabling environments for adaptive learning. We illustrate the implications of each lens for the *why*, *what* and *how* of measuring health system drivers across micro, meso and macro health systems levels, through three examples (digital health, maternal and perinatal death surveillance and review, and multisectoral action for adolescent health). Appreciating these underpinnings of measuring health systems and governance drivers of the health of women, children and adolescents is essential for a holistic learning and action agenda that engages a wider range of stakeholders, which includes, but also goes beyond, indicator-based measurement. Without a broadening of approaches to measurement and the types of research partnerships involved, continued investments in the health of women, children and adolescents will fall short.

**Summary box**

- By making explicit the different framings or lenses through which we see the health of women, children and adolescents, we make more transparent the choices made in terms of what is measured, why, how and for whom.
- Health systems measurement metrics to date largely focus on variables brought into view by the service delivery lens. However, both societal and systems lenses reveal important variables that are context specific and often intangible. These more intangible health systems drivers are subjective in nature and need joint interpretation by researchers and research participants.
- While cross-national governance and health metrics exist, they may be less useful for national-level policy-makers who are looking for more applied analysis of why, where and how to improve governance in health systems.
- A broader understanding of policy needs for advancing the health of women and children requires investing in a broader measurement agenda. This entails other research methodologies and methods, and also a reconsideration of the kinds of research partnerships constituted and how embedded they are with decision-makers who govern health systems at different levels for women’s, children’s and adolescents’ health.

## Introduction

Health systems play a critical role in improving and sustaining the health of women, children and adolescents by supporting intervention coverage and quality, promoting the rights of end users and intervening on the social determinants of health. Health systems consist of all the organisations, institutions, resources and people whose primary purpose is to promote and improve health.^1^

Key health systems inputs include human resources, financing, commodities, infrastructure and information systems to ensure high-quality health services. In addition to coordinating these inputs, health policy implementation and programme scale-up hinges on mobilising a multiplicity of actors for collective action to realise common goals within health programmes and across other sectors. This requires attention to people and how their relationships govern health systems across diverse contexts over time. These governance features, while less easily observable and often referred to as the underlying or ‘software’ of health systems, are key to understanding health systems performance and variation within and across jurisdictions.^2–4^ Governance is therefore not an additional building block of ancillary input in health systems, but the overarching frame within which the people, organisations, institutions and resources that make up health systems work.^5^ It is the force which binds or repels actors, relationships and resources across all levels of the health system to collectively realise health goals.

**Figure 1:**
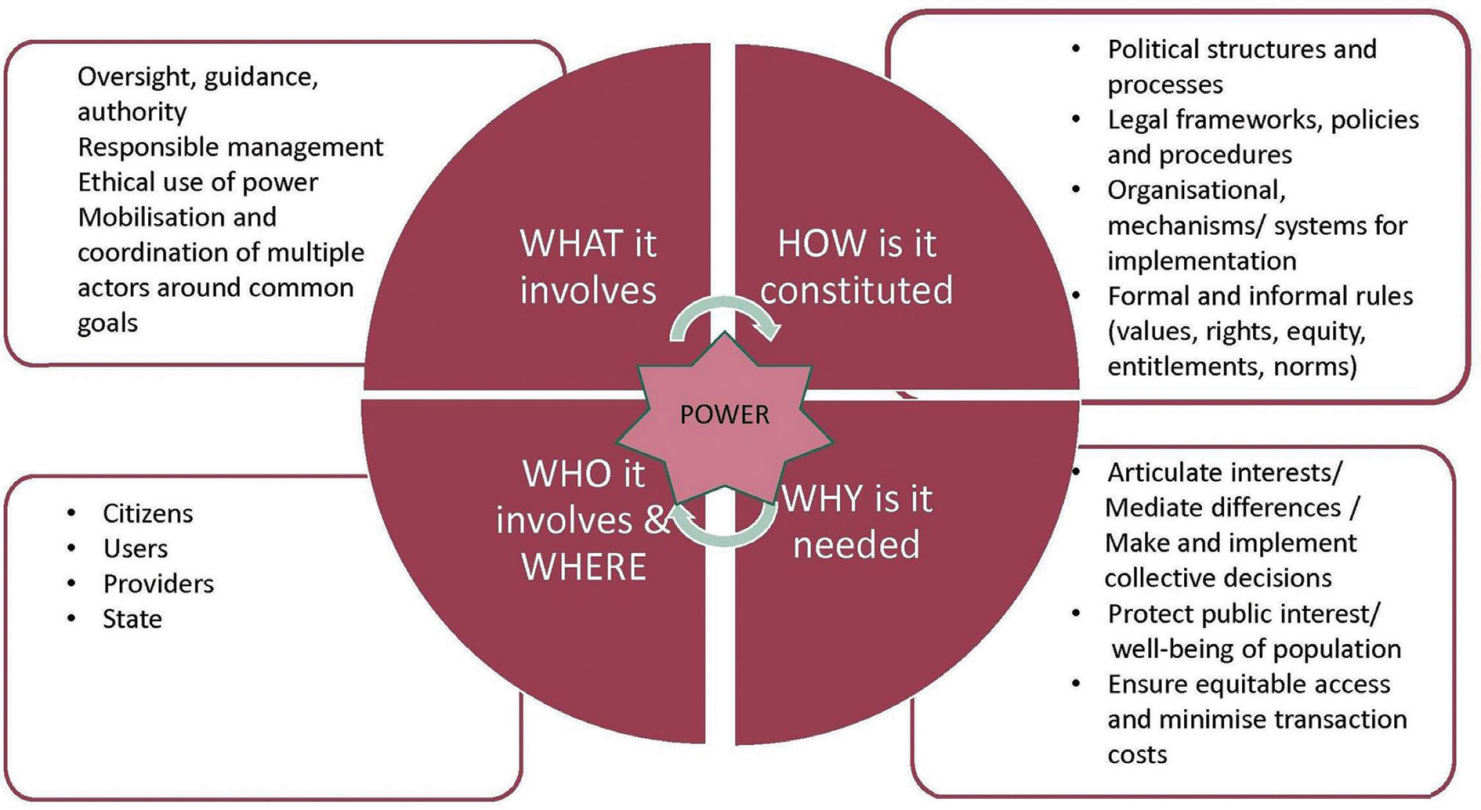
Elements of governance.

Governance involves the formal and informal rules and mechanisms that influence decision-making between citizens, providers, and the state in the public interest or not (figure 1). At its core, governance entails the mediation of power between diverse actors to influence the design and implementation of policies and services, although multiple definitions and frameworks for governance exist and continue to evolve, signalling its multidisciplinary origins.^5–8^

A governance perspective encourages taking a step back to understand how the health of women, children and adolescents is viewed before focusing on the micro-details of specific measurement metrics for health systems drivers. Three common framings or lenses include a *service delivery lens* aimed at scaling effective interventions, a *societal lens* oriented to empowering people with rights to effect change and a *systems lens* concerned with creating enabling environments for adaptive learning.

To illustrate this multidimensional view of health systems drivers, we apply these lenses to three examples. We use digital health to illustrate health systems dynamics relevant to governance at micro (individual level), maternal and perinatal death surveillance and response (MPDSR) to highlight facility-level meso (organisational level) dynamics, and multisectoral action for adolescent health for macro (structural level) dynamics.^9^

We conclude with reflections on the implications of these lenses and levels for measuring health systems drivers of the health of women, children and adolescents. The purpose of this methodology paper is not to propose universal measurements or indicators, but to develop understanding on how measuring health system drivers of the health of women, children and adolescents with a governance focus requires a broad approach to measurement, opening up our understanding of what we should be measuring, how, why and for whom. By doing so, we contribute to a more effective fulfilment of commitments to monitor progress in the health of women, children and adolescents in priority countries.

## Framing of women’s and children’s health: implications for why and what we measure

Political analysis engaged with understanding power relations and governance dynamics considers among other things how the framing of a problem, often unconsciously, shapes responses to it.^10^ In this paper, we argue that the framing of women’s, children’s and adolescents’ health is influenced by particular views of power^11^ and modes of governance,^12^ and that this framing in turn shapes how we understand health policy and programme implementation. This shapes our rationale or *why* we measure health systems drivers of women’s, children’s and adolescents’ health with a governance focus, but also *what* and *how* we measure these drivers (table 1).

**Table 1:** Why, what and how we measure health systems drivers of women’s, children’s and adolescents’ health with a governance focus

Why we measure	What we measure		How we measure	
Framing	Health systems drivers relevant to governance	Measurement variables	Research epistemologies, methodologies and methods	Research continuum
**Lens: Service delivery** **Focus:** Health conditions with effective interventions **View of power:** Technocratic **Mode of governance:** Hierarchical **Implementation focus:** Blueprints for what works	Policy mandate Coordination for continuity across levels and sectors Service delivery readiness User characteristics	Technical content of policies Management mechanisms: committees, review meetings, etc Inputs/resources (human resources for health, supplies, finances) User profile (literacy, gender, class, ethnicity, age)	**Epistemology:** Positivist **Methods:** Measuring adherence to recommended protocols (users and providers) Applying implementation checklists Analysing household, facility surveys or routine HMIS and system-generated data Ecological analysis of large datasets	**Disciplines:** Epidemiology, demography **Approach:** Replicable measurements that validate progress or highlight gaps **Evidence:** Cross-national data sets 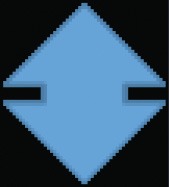 **Disciplines:** Multiple social science disciplines **Approach:** Context embedded research Reflective practice **Evidence:** Tacit and experiential knowledge
**Lens: Societal** **Focus:** People with rights **View of power:** Unidirectional/power over/zero-sum Co-produced/relational **Mode of governance:** Negotiating interests, enabling collaboration **Implementation focus:** Social processes/relationships	Macro: Political prioritisation Meso: Accountability dynamics Micro: Interpersonal dynamics supporting empowerment or marginalisation	Stakeholder positions and interests Participation/mobilisation Organisational cultures Transparency Credibility/trust Social capital and networks Social/informal norms Framing	**Epistemology:** Legal Pragmatic/constructivist **Methodologies:** Legal analysis Health policy and systems research **Methods:** Stakeholder analysis, political mapping Social network analysis Case study research	
**Lens: Systems** **Focus:** Complexity **View of power:** Creative: disruptive/productive **Mode of governance:** Principles enabling emergence **Implementation focus:** Interventions and people interact and evolve overtime	Dis/equilibria Feedback loops Eventuality of change Emergence Path dependence	Diversity of actors, varying power, alignment and interests Contextual permeability Adaptive or learning capacities Tipping points and motivation for emergence and change	**Epistemology:** Constructivist **Methodologies:** Ethnography Participatory action research Systems modelling **Methods:** Hidden transcripts Causal loop diagrams	

HMIS, health management and information systems.

### Service delivery lens

The dominant framing of the health of women, children and adolescents is of health conditions that have effective interventions. Implicit in this *service delivery lens* is a technocratic view of power that aims to separate science from politics and a mode of governance that is hierarchical assuming that authority flows from the top down. Implementation is therefore a matter of sequential inputs operationalised through predetermined blueprints or plans designed by experts from above into services to save lives.

From this service delivery lens, tangible health systems drivers that come into focus for measurement include policy mandates to support implementation (the availability of policies and their technical content), coordination to ensure continuity of care across levels and sectors (management mechanisms), service delivery readiness (health system building block inputs and resources) and user characteristics (gender, class, ethnicity, age, geography, etc). The later variable enables one to have a baseline to assess various equity dimensions of service delivery.

### Societal lens

A different and equally compelling framing of women’s, children’s and adolescents’ health is of the people involved and their rights shaped by the societies in which they are embedded. Within this societal lens, a legal rights–based perspective can view power as a series of zero-sum bargains or binary pairings. It therefore seeks to clarify roles and responsibilities, as well as entitlements of rights claimants and obligations of rights holders. A health policy and systems perspective, informed by a view of power that is co-produced or relational, examines the negotiations and discretion of communities, healthcare users, health workers and/or managers to buy in to reforms or resist them. Both perspectives view implementation as a social process dependent on social context and the relationships brokered between diverse health system actors.

From a societal lens, there is not a universal checklist of tangible factors to measure, but rather a range of phenomena that can be measured depending on the issue, context and health system level. Three illustrative health systems drivers for women’s, children’s and adolescent’s health within this framing are those related to (1) political prioritisation at the macro-level of societies, (2) accountability dynamics within the meso-level of organisations and (3) how interpersonal dynamics between individuals at the micro-level of health systems support empowerment or marginalisation. Measurement, depending on the issues at hand, would focus on, for example, the following intangible variables: actor interests and social networks; organisational cultures and trust; social capital; framing and social norms. These variables help to understand whether the interests and capabilities of marginalised actors are considered and transformed or whether power structures continue to disenfranchise them. These are vital to understand how and why inequalities persist and how they can be transformed.

### Systems lens

As understanding of the social relationships that underpin implementation has deepened, so has an appreciation for the complexity^13^ and fluidity with which interventions and people dynamically interact and evolve over time through systems in anticipated, but also unanticipated ways. Implicit in this systems lens is a view of power as creative, which can be directed in disruptive and productive ways. Governance approached through this lens recognises that power is dispersed in organisations, and that planned change interfaces with spontaneous forms of self-organisation referred to as emergence. Enabling positive forms of emergence is key to governance and in this regard, implementation is aided by understanding current system dynamics, and the role of equilibria and feedback loops. Varying degrees of cooperation or contestation, and the overall effects of positive and negative feedback loops establish path dependence or the emergence of new equilibria in systems. Key dimensions to measure for policy implementation are the diversity of the actors involved and their dynamic interdependence, their adaptive learning capacities, the permeability of context, and key triggers or tipping points that motivate and support emergence or change.^14–17^ This perspective enables us to understand key triggers and alignment of actors that support more equitable change, but also the backlash against such social change.

These framings and corresponding lenses are not mutually exclusive or competing paradigms, but rather ways of looking at different dimensions of women’s, children’s and adolescents’ health, which taken together provide a holistic understanding of health systems drivers. We explain them further with a focus on governance through examples in the next section.

## What to measure across lenses and levels: illustrative examples from women’s, children’s and adolescent’s health

Given that governance and health systems relationships are pervasive contextual drivers of women’s, children’s and adolescents’ health, we use three examples to illustrate the possibilities of what to measure across lenses and levels (tables 2–4). These examples represent key interfaces between people in health systems and can be analysed at micro (individual), meso (organisational) and macro (structural) levels. Each of these examples, while initiated at different levels of the health system, can be seen as governance interventions potentially disrupting and recreating relations across health system levels.

**Table 2:** Lenses and levels for examining drivers of digital health

Health systems drivers with a governance focus	Health system levels		
	Macro	Meso	Micro
Service delivery lens			
Policy mandate Coordination mechanisms Service delivery readiness User capacity	Policies on privacy of personal data, interoperability, procurement, etc Network coverage: cell phone towers	Composition and organisational location of task force Technology design choices Network coverage: mobile network operator verification systems Health worker workload Financial resources to support the programme	User mobile literacy, access and ownership Network coverage: SIM turnover, handset type and functionality
Society lens			
Political prioritisation Accountability dynamics Interpersonal dynamics	Trust in government or private companies maintaining information responsibly	Incentives and positionality of implementing partners (Ministry of Health, technology partners, mobile network operators, academic/research partners) Stakeholder relationships: NGOs with prior positive relationships with government more able to present data with negative findings to government Health worker responses and prioritisation	Women with culture of concealing pregnancy, not being aware that they would be receiving SMS, can distrust or be jeopardised by text messages from unknown numbers DRC: more men than women accessing digital app on family planning originally targeted for women; is male power reinforced vs transformed?^23^
Systems lens			
Dis/equilibria Feedback loops Eventuality of change Emergence Path dependence (Dynamics can link across micro, meso and macro levels)	Tanzania: trained enumerators using smartphone apps in people’s homes was a trigger for conversations and relationship building… community validation meetings where people discussed results offline and local health workers present who saw it as an opportunity to channel demands upwards to district authorities for resource allocation decisions^24^ South Africa: health workers adapting registration processes from individual to batch registration; increases numbers of people registered, decreases waiting time for services, but uncertain consent procedures^83^ Nigeria: women promised recharge cards to elicit participation, but then not all tech partners agreed, backfired against women who responded but belonged to these excluded tech partner networks… women then deleted messages and refused to participate^24^		

DRC, Democratic Republic of Congo; NGO, non-governmental organisation.

**Table 3:** Lenses and levels for examining drivers of maternal and perinatal death surveillance and response (MPDSR)

Health systems drivers with a governance focus	Health system levels		
	Macro	Meso	Micro
Service delivery lens			
Policy mandate Coordination mechanisms Service delivery readiness User capacity	National MPDSR policy and guidelines Death notification requirements (legal framework for notifying deaths) Legal mandate to involve communities and other sectors Human resources shortages across the system but particularly for maternal and child health specialists	Committees formed Committee composition: profession, gender, seniority Meeting frequency Publication of proceedings Strategy for staff orientation to MPDSR Availability of MPDSR tools Health worker workload Functionality of information systems	Competencies of managers, supervisors, providers to analysis and interpret data and information
Society lens			
Political prioritisation Accountability dynamics Interpersonal dynamics	National prioritisation of preventing maternal and perinatal deaths Perceived preventability of deaths Social implications of political party affiliation, gender, class, among committee members, etc Community engagement	Leadership: individuals (champions) and of system (space for teamwork) Moving away from blame to learning environment/ trust Credibility of HMIS system Visibility of effect/impact Health worker responses and prioritisation	Confidence of and capability of health workers to complete and analyse deaths Relationship between committee members Mentorship, clinical outreach and supervisory activities through district engagement
Systems lens			
Dis/equilibria Feedback loops Eventuality of change Emergence (Dynamics can link across micro, meso and macro levels)	Kenya: MPDSR process/outcomes fail to deliver on actions due to health system barriers which perpetuates a demoralising work environment and undermines commitment to attending meetings^35^ Nigeria: improved MPDSR led to increased reporting of deaths and therefore an increase in mortality further documenting poor performance. However, responses to insufficient blood supply led to community mobilisation for blood donor club formation. Inclusion of findings in State Medium Term Strategy led to the provision and maintenance of blood banks in state hospitals^36^		

HMIS, health management and information systems.

**Table 4:** Lenses and levels for examining drivers of multisectoral action for adolescent health

Health systems drivers with a governance focus	Health system levels		
	Macro	Meso	Micro
Service delivery lens			
Policy mandate Coordination mechanisms Service delivery readiness User capacity	Policies across different sectors recognising adolescent health (Adolescent Health in All Policies approach)^84^ Policies across different sectors recognising multisectoral action for adolescent health	Existence of adolescent health committee/unit^85^ Constitution and functioning of committee/unit Location and linkages of the committee/unit within sectoral hierarchies Availability and authority to deploy resources	Profile of policy champions: profession, seniority, age, gender Competency of all stakeholders in adolescent health and multisectoral actions Capacity to generate and use evidence on mutlisectoral action^86^
Society lens			
Political prioritisation Accountability dynamics Interpersonal dynamics	Adolescent leadership and participation and mobilisation overall Social determinants of health including, gender, diversity and socioeconomic and political context Framing and alignments of sector goals	Incentives and constraints of stakeholders, including adolescents Leadership and organisational cultures supporting multisectoral action^40^	Social networks and histories between policy advocates Trust, communication and credibility between policy advocates
Systems lens			
Dis/equilibria Feedback loops Eventuality of change Emergence (Dynamics can link across micro, meso and macro levels)	Policies that prohibit adolescent girls from being pregnant while being in school, inhibits early care seeking for pregnancy care by these adolescents and has a negative impact on their future education and health^38^ Not including adolescents in leadership and participation during the design, implementation, monitoring and evaluation of adolescent health programme will contribute to a negative feedback loop in terms of nature, quality and impact of the programme^87^ Initial gains on collaborating on health education or HPV at schools, builds trust and relationships between sectors, that enables further work on more complex mutual aims such as mental health or comprehensive sexual and reproductive health programmes or violence prevention^38^		

HPV, human papilloma virus.

### Digital health

Digital health entails the use of digital, mobile and wireless technologies in support of health, with the potential to link users, providers and managers in new ways to improve uptake, quality and continuity of care. Despite its promise, few digital health solutions are successfully scaled in low-income and middle-income countries, including those addressing health governance.^18^ The reasons underpinning this are technological and social, encompassing resource constraints but also governance challenges.^19–21^

We map what health systems drivers to measure in digital health through service delivery, society and systems lenses in table 2. When we focus on user experiences at the micro-level interpersonal interface of health systems, a service delivery lens is essential to measure uptake of digital health interventions.^18 22–25^ Moving beyond this descriptive level, a societal and systems lens enables us to understand the social barriers to accessing digital health,^26 27^ as well as the social implications of such access. These additional lenses allow us to move beyond measuring whether or if digital health is being used, to understanding how and why end users engage with the innovation and how they adapt them in diverse contexts over time.^24 27 28^

### Maternal and perinatal death surveillance and response

Moving from micro-level interpersonal interfaces aided by digital health to meso-level dynamics at facility level, a key quality improvement initiative is MPDSR as it supports review, learning and corrective action among diverse stakeholders responsible for providing services. MPDSR is the process of capturing information on the number and causes of deaths and then undertaking systematic, critical analysis of the quality of care received, in a no-blame, interdisciplinary setting, to develop and implement responses to prevent future deaths.^29–31^ Favourable policies for MPDSR are in place,^32^ yet few countries have robust operational MPDSR systems,^32^ and the likelihood for improvement only occurs if the audit cycle is fully implemented.^31^

We examine the health systems drivers of MPDSR through service delivery, societal and systems lenses and across health system levels (table 3), but focus on the meso-level here. At the meso-level or organisational level of health systems, a service delivery lens prioritises measurements of tangible markers of MPDSR implementation, such as number of meetings, dissemination of proceedings, number of trainings, use of guidelines, workload of MPDSR committee members and so on. In addition, a societal lens would examine issues of trust, credibility and hierarchies between MPDSR stakeholders.^33 34^ A systems lens would look at how MPDSR triggers responses across health system levels, unleashing further scrutiny or resources, that would either empower or further demoralise health workers depending on how feedback loops are managed.^35 36^

### Multisectoral action for adolescent health

At a macro-level, the health sector works with sectors outside of those directed by ministries of health, depending on their alignment to address key social determinants. Calls for multisectoral action for adolescent health are frequently made, given that many of the determinants of the social and health inequalities faced by adolescents lie beyond the remit and resources of the health sector.^37^ Yet beyond scattered projects, few examples of sustained and robust multisectoral action for adolescent health exist. Only a few service delivery school-based examples exist where health supports work led by the education sector.^38^ Despite consensus on the rationale for multisectoral action for health dating back to Alma Ata,^39^ challenges realising it are not purely technical or programmatic, but rather relate to governance.^40 41^

While all health systems drivers by lenses and levels are detailed in table 4, here we focus on macro-level drivers of multisectoral action for adolescent health. At a macro-level, a service delivery lens would count the existence of multisectoral policies and alignment of policies across different sectors. For example, school policies that expel adolescent girls from school for being pregnant do not align with health policies that aim to provide services to them. Marketing to adolescents by transnational corporations of alcohol, smoking and fast foods may align with trade policy, but not with health policy. A societal lens helps to assess whether the existence of policies is likely to lead to action by understanding the intangible workings of political alliances and constituencies involved and whether they share a common framing motivating multisectoral action. Lastly, a systems lens would look at the history of these relationships to see whether adaptive learning supports ongoing trust, social capital and collaboration across sectors overtime, as detailed in the examples in table 4 below.

## Lenses and levels for understanding health systems drivers with a governance focus: implications for how we measure and for whom

Numerous efforts to measure governance and health have evolved reflecting different disciplinary origins, research groups and policy audiences. Ecological analysis has linked good governance indicators with better health outcomes usually by improving the effectiveness of public health spending.^2 42–45^ Initiatives have also developed frameworks or indicators to measure health system governance.^46–48^ While these metrics enable cross-national comparisons that are useful for donors and international organisations, they may be less useful for national-level policy-makers who are looking for more applied analysis of why, where and how to improve governance in health systems^5^ so that they deliver for those most likely to be left behind.

Understanding and responding to broader policy needs entails moving towards a broader range of disciplinary approaches in research on governance in health systems. Embedded research partnerships that can grasp historical and sociological contexts, and that facilitate co-production of emergent understandings of governance grounded in the programmatic realities of responding to women’s, children’s and adolescents’ health and rights are critical. This entails building capacities of local research organisations, as well as the research affinities of those running health programmes.

Cross-national comparisons in measuring progress for women’s, children’s and adolescents’ health relies on replicable measurements standardised across large numbers of jurisdictions (nation states, subnational districts). These are appropriate and effective for measuring the directly observable health systems drivers seen from a service delivery lens. However, many of the health systems drivers seen from a society lens are less easily observable aspects of human relationships that require research methods that enable a more in depth understanding of the social and system dynamics involved. These more intangible health systems drivers are subjective in nature and need joint interpretation by researchers and research participants. The corresponding context-specific embeddedness quality of such research necessitates research partnerships that are fostered over time beyond any data point or research study.

Understanding and reflecting on the different framings of women’s, children’s and adolescents’ health and their corresponding service delivery, societal and systems lenses across health system levels enables an appreciation of the broad range of epistemologies and the continuum of research approaches^49 50^ that should be embraced to measure health systems drivers appropriately and effectively. A crucial first step towards that direction entails recognising the different epistemologies, or theories of knowledge, that underline the types of measurement that support understanding of health systems drivers identified by service delivery, societal or system lenses. We conclude this section by outlining these different epistemologies and the range of studies that can be supported depending on the specific lens and health issue focused on.

From a service delivery lens, the underlying epistemology is positivist. Illustrative examples of descriptive studies from this lens and research perspective include policy surveys of integrated Community Case Management (iCCM), infant and young child feeding^51 52^ and maternal death surveillance and response^36^; iCCM health systems indicators^53^ and various efforts to measure implementation strength^54–56^; and readiness to scale newborn survival interventions^57^ or, in the case of digital health, technical functionality.^26^ These studies provide valuable descriptive baselines of the technical content of policies and the readiness to implement them, often through objectively verifiable measurements. Other positivist studies also assess impact of interventions, even quite complex social interventions such as social accountability^58^ or women’s groups.^59^ They mostly do not, however, explain how and why policies and interventions are implemented or why they work. Other methodologies are better suited to reveal the subjective and complex nature of consensus building, ownership or internalisation among diverse stakeholders needed to commit to the extensive collaboration and collective action required to sustain interventions and policy implementation overtime.^15^

From a society lens, the research approach tends to be pragmatic or constructivist. It includes health policy and systems research that examines health systems as context specific, constituted by people and their relationships imbued with symbolic meaning that is not always visible or observable. Illustrative examples include studies that examine macro-level political influences such as the prioritisation of maternal and newborn health^60–62^ or donor practices in global health.^63–65^ At the meso-level, evaluations have unpacked accountability dynamics in maternal and child health services^33 58 66–70^ or social network influences over provider behaviour.^71 72^ Recent attention to the disrespect and abuse of women seeking obstetric services and the perspectives of providers involved in such care has highlighted issues of trust, social norms and organisational culture as central to the patient–provider dynamics at micro and meso levels.^73–76^ Many of these studies include mixed methods or case study research approaches.

From a systems lens, the underlying epistemology tends to be constructivist, although the methodologies and methods of this approach range from ethnography and participatory action research to causal loop analysis, systems dynamic modelling and network analysis. Applications of this perspective include the sustainability of iCCM in Rwanda^77^ or urban health programmes for maternal and child health in Bangladesh,^78^ the drivers of coverage and governance of immunisation programmes in India,^79^ and unforeseen resistance to global policy on male circumcision in Malawi^80^ or to global HIV funding mechanisms in India.^81^ These analyses are highly context dependent and often reflect embedded research approaches, where implementers collaborate with researchers iteratively reflecting on practice and building on tacit and experiential knowledge.^82^

## Conclusion

Health systems drivers are key to understanding the enabling factors, social dynamics and rights that underpin coverage and equity of women’s, children’s and adolescents’ health. These drivers are better understood when seen through service delivery, society and systems lenses. These lenses reveal complementary but distinct health system drivers that together explain health system performance. While progress has been made in developing tools for describing these drivers from a service delivery lens, further understanding of the less observable elements that shape human behaviour in health systems identified by society and systems lenses is needed. This entails other research methodologies and methods, and also a reconsideration of what kinds of research teams are constituted and how embedded they are with decision-makers who govern health systems at different levels for women’s, children’s and adolescents’ health.
